# Probiotic *Lacticaseibacillus rhamnosus* GG Increased Longevity and Resistance Against Foodborne Pathogens in *Caenorhabditis elegans* by Regulating MicroRNA *miR-34*

**DOI:** 10.3389/fcimb.2021.819328

**Published:** 2022-01-19

**Authors:** Bohyun Yun, Sangdon Ryu, Minkyoung Kang, Juyeon Lee, Jiseon Yoo, Younghoon Kim, Sangnam Oh

**Affiliations:** ^1^ Department of Functional Food and Biotechnology, Jeonju University, Jeonju, South Korea; ^2^ Department of Agricultural Biotechnology and Research Institute of Agriculture and Life Science, Seoul National University, Seoul, South Korea

**Keywords:** *C. elegans*, probiotics, *L. rhamnosus* GG, microRNA, immunity

## Abstract

In this study, we investigated the relation of probiotic activity of *Lacticaseibacillus rhamnosus* strain GG (LGG) and expression of microRNA to immune response and longevity in *Caenorhabditis elegans* host model. First, we evaluated the survival rate of *C. elegans* due to LGG exposure and bacterial colonization in the intestine. Next, the expression of mRNA and miRNA was analyzed in *C. elegans* exposure to LGG for 24 h using microarray. After exposure to LGG to *C. elegans*, colonized LGG was observed in the intestines of *C. elegans* and induced to extend lifespan. Moreover, persistent LGG in the intestine significantly enhanced the resistance of *C. elegans* exposed to both pathogenic bacteria and prolonged the lifespan of *C. elegans*. Transcriptome analysis indicated that LGG affected the expression levels of genes related to the innate immune response and upregulated the abundance of genes in multiple pathways of *C. elegans*, including Wnt signaling, TGF-beta signaling and mitogen-activated protein kinase (MAPK) pathways. In addition, qRT-PCR analysis confirmed that the expression of antibacterial genes was increased by LGG. Moreover, as the expression of microRNA *miR-34* and immune-related pathways increased by exposure to LGG, the lifespan of *C. elegans* increased. However, in the *miR-34* mutant *C. elegans*, the lifespan by LGG did not increase, so it was determined that *miR-34* indirectly affects immune-related pathways. There was no significant difference in the expression of PMK-1 for LGG exposure in miR-34 mutants, suggesting that miR-34 may regulate PMK-1. In conclusion, we suggest that exposure of LGG to *C. elegans* enhances lifespan and resistance to food-borne pathogen infection by stimulating *miR-34* and indirectly promoting PMK-1 activity.

## Introduction

*Lacticaseibacillus rhamnosus* strain GG (LGG) is one of the most widely studied probiotic strains for dairy foods including yogurt and cheese, and is generally recognized as safe (GRAS) by the Food and Drug Administration (Groele et al., 2017). LGG, originally isolated from human feces by Gorbach and Goldin (Segers and Lebeer, 2014), is an ideal probiotic that is resistant to stomach acid and bile, so it can survive the gastrointestinal tract, has the ability to consistently implant human intestinal epithelial cells and colonize the gut, produces an antimicrobial substance, has a rapid growth rate, and has beneficial effects on health (Doron et al., 2005). In addition, LGG has been applied to various disease states in clinical trials and has shown many benefits to the host, including improvements in diarrhea (Arvola et al., 1999; Basu et al., 2009), atopic diseases (Kalliomäki et al., 2003), anti-obesity (Luoto et al., 2010) and respiratory pathology (Davidson et al., 2011). Recently, LGG has been reported to have a beneficial effect on the host by restoring the intestinal flora imbalance caused by disease and maintaining intestinal homeostasis (Durack et al., 2017; Zhang et al., 2018; Han et al., 2019). Yan et al. reported that the LGG-derived protein p40 prevents and treats colon epithelial cell damage and inflammation in a colitis model (Yan et al., 2011). In addition, Lin et al. reported that LGG can protect against deoxynivalenol-induced bowel damage by regulating the intestinal microflora to promote the production of butyrate and thereby inhibit the IRE1a/XBP1 signaling pathway (Lin et al., 2020). In particular, LGG can reduce the risk of colon cancer by regulating the intestinal microflora and downregulating pro-inflammatory molecules (Gamallat et al., 2019).

Importantly, probiotic strains have been reported to be effective in gut health by regulating microRNA expression (Chen et al., 2017; Heydari et al., 2019). MicroRNAs (miRNAs) are small noncoding RNA molecules of approximately 20-22 nucleotides (Desvignes et al., 2019). miRNA controls gene expression *via* base complementarity between the seed region of miRNA and the 3’-untranslated region (UTP) of the target mRNA (Allegra et al., 2020). In many cells, miRNAs have been reported to regulate pro- and anti-inflammatory responses to maintain homeostasis of the host (Behrouzi et al., 2020) and are also involved in innate and adaptive immunity (Lindsay, 2008; Garo and Murugaiyan, 2016). In addition, commensal bacteria in the gut are involved in host health and disease, and miRNAs have been shown to modulate human-microbial interactions in the host gut (Behrouzi et al., 2020). Unfortunately, when the literature was reviewed, there had been no previous studies in which LGG regulates miRNA expression in the host. *Caenorhabditis elegans* is known as a suitable experimental model for host microbiota studies (Cabreiro and Gems, 2013). In particular, the innate immune system, the first line of defense against microbial infections, is the first and phylogenetically oldest line of defense in all multicellular organisms (Isailovic et al., 2015). Research on *C. elegans*, a genetically tractable model, has provided valuable insight into the functional aspects of antiaging and innate immunity and has greatly contributed to our understanding of the multiple genetic pathways that determine longevity (Ikeda et al., 2007; Irazoqui et al., 2010; Park et al., 2018). The mechanisms of microbial etiology, host defense and the role of probiotics in *C. elegans* have previously been reviewed (Mylonakis and Aballay, 2005; Kim and Mylonakis, 2012; Pukkila-Worley and Ausubel, 2012; Balla and Troemel, 2013; Komura et al., 2013; Park et al., 2018). Recent studies have reported that miRNAs play an important role in this mechanism (Dai et al., 2015; Isik et al., 2016; Xiong et al., 2016). In particular, studies using *C. elegans* as a mechanism related to miRNAs have been reported (Zhou et al., 2019; Li et al., 2020). The purpose of this study was to demonstrate the longevity and resistance of *C. elegans* exposed to LGG to pathogenic bacterial infections. We also aimed to demonstrate that LGG can have beneficial effects on a host by regulating immunity-related genes or miRNAs in the host.

## Materials and Methods

### Bacterial Strains and *C. elegans* Growth Conditions

*L. rhamnosus* strain GG (ATCC 53103), *Salmonella* Typhimurium SL1344, *Escherichia coli* OP50, *Staphylococcus aureus* RN6390 and *Enterococcus faecalis* MMH594 were grown at 37°C using De Man, Rogosa, and Sharpe (MRS; Difco, Detroit, MI) broth for LGG, Luria-Bertani (LB; Difco) medium for *S. Typhimurium* SL1344 and *E. coli* OP50, tryptic soy broth (TSB; Difco) for *S. aureus* RN6390, and brain heart infusion (BHI; Difco) for *E. faecalis* MMH594. For long-term storage, cultures were maintained at −80°C with 15% glycerol. All strains were sub-cultured twice prior to experimental analysis. To prepare live bacterial lawns for *C. elegans* feeding, after the bacteria were grown in each growth medium and washed five times with M9 medium, 500 μl of each type of cell (2.0 × 10^9^ CFU/ml) was spread on Nematode Growth Medium (NGM) plates and dried at room temperature for 3 h. Each live bacterial lawn was stored at 4°C and used within a week.

*C. elegans* N2 Bristol wild-type, CF512 *fer-15*(*b26*)*II*;*fem-1*(*hc17*)*IV* strains were used in this study. *C. elegans* were routinely maintained in NGM agar inoculated with OP50, an internationally established feed using standard techniques (Park et al., 2018). Eggs were extracted from sodium hypochlorite-sodium hydroxide solution (1.0%–1.3% w/v sodium hypochlorite, 500 mM sodium hydroxide) from egg-bearing *C. elegans* to obtain L1 *C. elegans* and then transferred to NGM plates seeded with OP50. Synchronized L1 *C. elegans* were grown at 25°C to obtain L4/young adult *C. elegans*.

### *C. elegans* Longevity Assay and Killing Assay

*C. elegans* longevity was assessed as previously described method (Kim and Mylonakis, 2012; Park et al., 2018). Briefly, 100 μl aliquots of concentrated bacteria (2.0 × 10^9^ CFU/ml) were plated on 35-mm NGM agar plates, and L4/young adult *C. elegans* of N2 or mutant *C. elegans* were individually transferred with a platinum wire onto OP50 or LGG plates and maintained at 25°C. The numbers of live *C. elegans* were measured daily for viability using a dissection microscope (Olympus SZ40, Olympus, Japan). Live *C. elegans* were transferred to fresh plates daily during the progeny production period and, thereafter, were transferred every 3 days. *C. elegans* was determined to be dead if it was not moved by gentle touching with a platinum wire pick. In addition, for *C. elegans* killing analysis, L4/young adult *C. elegans* were placed on plates exposed to LGG or OP50 for 24 h at 25°C. *C. elegans* pre-exposed to LGG or OP50 were then transferred to NGM plates plated with pathogenic bacteria (*S.* Typhimurium SL1344, *S. aureus* RN6390 and *E. faecalis* MMH594) and examined at 24 h intervals until all the *C. elegans* died at 25°C.

### Bacterial Attachment Assay in *C. elegans* Intestinal Tract

The bacterial attachment assay was performed according to an established method (Kim and Mylonakis, 2012; Park et al., 2018) with some modifications. LGG attachment assay in the *C. elegans* intestinal tract collected *C. elegans* after 24 h of exposure to LGG lawn prepared on NGM agar plates. Analysis of *E. faecalis* attachment in *C. elegans* intestinal tracts was exposed to LGG lawns for 24 h and then transferred to *E. faecalis* lawns. *C. elegans* were then collected on 0, 1, 3 and 5 days. Collected *C. elegans* were washed five times with M9 buffer and transferred to BHI plates containing kanamycin (100 μg/ml) and streptomycin (100 μg/ml). *C. elegans* were washed with 5 μL drops of gentamicin solution (25 μg/ml) for 5 min to remove attached bacteria from the body of the *C. elegans*. After removing surface bacteria, *C. elegans* were washed five times with M9 buffer, and *C. elegans* were placed in new sterile tubes containing M9 buffer with 1% Triton X-100 and mechanically disrupted by using a mortar and pestle (Kontes, Vineland, NJ, USA). *C. elegans* lysates were serially diluted in M9 buffer, which was plated on BHI agar containing 80 μg/ml kanamycin for *E. faecalis* or modified MRS (pH 5.0) agar for LGG. Plates were incubated at 37°C for 24 h. Colonies were quantified and used to calculate the number of bacteria per *C. elegans*.

### Transmission Electron Microscopy (TEM) in the *C. elegans* Intestinal Tract

For microscopic observation, *C. elegans* were quickly placed on 2% agarose pads with drops of 10 mM NaN_3_ in M9 butter. *C. elegans* were observed under a microscope (Nikon Eclipse Ts2, Nikon, Japan). Simultaneously, bacterial attachment was visualized by TEM (Hitachi H-7650, Hitachi High Technologies, Japan) to determine their persistence on the *C. elegans* intestinal epithelium. *C. elegans* were plated on 60-mm NGM plates seeded with LGG or control plates seeded with OP50. Observations for each sample were evaluated for at least 10 cross sections, and representative images were selected.

### Transcriptome Analysis for mRNA and miRNA Expression

Microarray experiments were performed to compare the expression of mRNA and miRNA with OP50 when *C. elegans* was exposed to LGG for 24 h. *C. elegans* were collected through M9 buffer after 24 h exposure to LGG or OP50, washed three times, and pelleted by centrifugation for RNA isolation. After centrifugation, total RNA was isolated from *C. elegans* using QlAzol (QIAGEN, Germany) and RNeasy mini kit (Qiagen, *Valencia*, *CA*) according to the manufacturer’s instructions. RNA was quantified using a 2100 Bioanalyzer (Agilent Technologies, Santa Clara, CA, USA) with an RNA 6000 nano kit. Targeted microarray analysis for mRNA and miRNA was performed by a commercial company (eBiogen, Seoul, Korea) using a *C. elegans* GE Microarray 4x44K (Agilent) and Affymetrix GeneChip_miRNA arrays (Affymetrix), respectively. A gene was considered differentially expressed when the p value for comparing chips was lower than 0.05 (to assure that the change in gene expression was statistically significant and that false positives arose less than 5%). The differential gene expression data have been deposited in the NCBI Gene Expression Omnibus (http://www.ncbi.nlm.nih.gov/geo/) and are accessible through Accession No. GSE190701.

Cluster analysis was based on protein functional enrichment by DAVID Bioinformatics Resources 6.8. Cluster analysis based on functional enrichment was used to study potential connections and differences in specific functions (KEGG pathways and protein domains). Functional classification information and corresponding P-values were collected, and functional classes were screened in one or more protein clusters. The Z-transformed dataset was analyzed by hierarchical clustering (Euclidean distance, complete connected clustering). The clustering association was visualized using heatmap.2 in the R package (v3.6.3).

### qRT-PCR Validation

Total RNA from *C. elegans* was quickly isolated following the protocol of TRIzol reagent (Invitrogen, USA) and purified using the RNeasy minikit (Qiagen, Germany). After RNA isolation, 50 ng of total RNA was used for qRT-PCR using the SuperScript III Platinum SYBR green one-step qRT-PCR kit (Invitrogen, USA). qRT-PCR was performed using Applied Biosystems StepOnePlus Real-Time PCR Systems (Applied Biosystems, USA). Primer validation and qRT-PCR were performed as described previously (Kim and Mylonakis, 2012). Relative expression levels were calculated using the 2^−ΔΔCT^ threshold cycle method (Livak and Schmittgen, 2001), and the control gene *snb-1* was used to normalize the gene expression data (Irazoqui et al., 2008).

### Immunoblot Analysis

Immunoblot analysis was performed according to an established method (Jeong et al., 2018) with some modifications. Briefly, *C. elegans* was exposed to LGG and OP50 for 24 h. After treatment, the *C. elegans* were washed with M9 buffer and homogenized in cold PBS supplemented with protease and phosphatase inhibitor cocktail (Thermo Scientific, USA). The residues in the lysates were removed by centrifugation at 800 × g for 10 min at 4°C, and the protein content was measured. Approximately 30 µg of protein was loaded onto 12% reducing SDS-acrylamide gels and electrophoresed and transferred to a polyvinylidene fluoride (PVDF) membrane with a 0.2 μm pore size (Bio-Rad Lab, USA). After transfer, the membranes were blocked with 5% skimmed milk in Tris-buffered saline containing 0.1% Tween 20 (TBST) for 2 h. After blocking in 5% skimmed milk in TBST, the membranes were incubated for 1 h with primary antibodies [phospho-PMK-1 (Cell Signaling) or β-actin (Santa Cruz)] diluted in TBST with 5% skimmed milk at RT, followed by incubation with the secondary antibodies anti-rabbit IgG (Thermo Fisher) or anti-mouse IgG (Santa Cruz) at RT for 1 h. HRP signals were visualized using chemiluminescence (ECL kit, Amersham) and an image analyzer (C-DiGit^®^ Blot Scanner, Li-COR).

### Statistical Analysis

*C. elegans* survival analysis was performed using the Kaplan–Meier method, and the significance of differences between survival curves was determined using the log-rank test (STATA6; STATA, College Station, TX, USA). Student’s *t*-test was performed to determine significant differences for CFU counting for determining bacterial abundance. The data shown are presented as the means ± SEM of three or more independent experiments, and the differences were considered statistically significant at *p*<0.05 using Student’s *t*-test.

## Results and Discussion

### LGG Is Not Harmful to the *C. elegans* Host and Colonizes the Intestine of the Host

**Figure 1:**
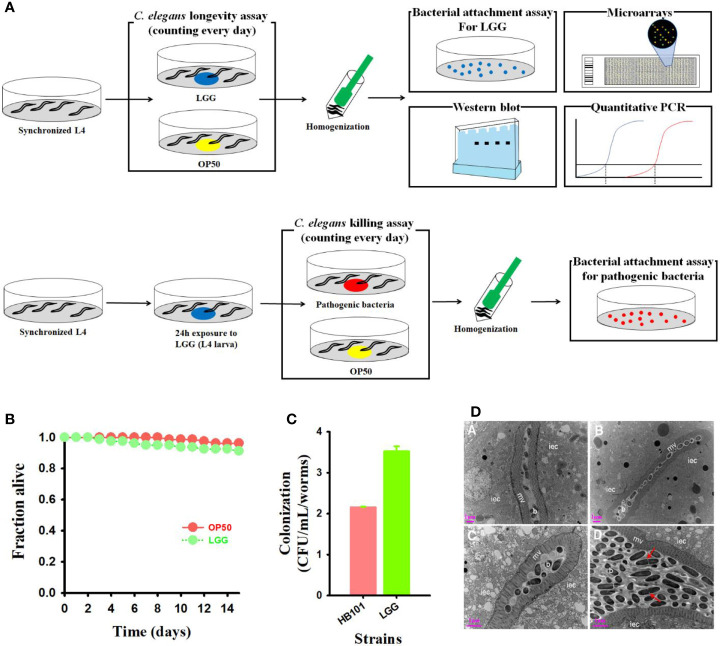
Treatment of probiotics LGG to *C. elegans* and high adhesion in the intestine. **(A)** Schematic diagram of experimental design (Details are in materials and methods). **(B)** Monitoring the viability of *C. elegans fer-15;fem-1 C. elegans* (n=40 per well) exposed to LGG or OP50 (normal feed). **(C)** Bacterial counts (colony forming unit) in *C. elegans* intestine and **(D)** TEM images of transverse mid-body sections of C. elegans fed with OP50 (a, b) or LGG (c, d). Higher magnification showing microvilli, intestinal epithelial cells, bacterial colonization in the intestinal lumen iec, intestinal epithelial cell; mv, microvilli; b, bacterial cells (OP50 or LGG). Scale bar = 1 μm.

We performed toxicity studies to evaluate whether LGG, a well-known probiotic (Capurso, 2019), is harmful to *C. elegans* hosts. To this end, lifespan analyses were performed by monitoring the LGG-fed *C. elegans*, starting at the L4 larval stage, compared to the control *C. elegans* exposed to the nonpathogenic prey *E. coli* OP50 or probiotic strain LGG (**Figure 1A**). We found no significant difference in the lifespan of *C. elegans* exposed to OP50 and LGG in *C. elegans* (**Figure 1B**). Hence, we concluded that LGG was not toxic to *C. elegans*.

LGG contains two pilus gene clusters, *SpaCBA* and *SpaFED*, which play a beneficial role in the host due to its intestinal attachment ability (Guerin et al., 2016). Intestinal cells of *C. elegans* are similar in structure to human intestinal cells and are often used to determine the defense mechanisms of the intestines of the host (Park et al., 2018). Therefore, we assessed adhesion ability by exposing LGG to *C. elegans* to confirm the attachment of LGG. LGG showed high colonization ability and an approximately 1.6-fold higher CFU count than OP50 (**Figure 1C**). Similar to the plate number, the intestine of the *C. elegans* was completely colonized with LGG after feeding LGG from the *C. elegans*, and LGG was not digested as a cell mass surrounded by an extracellular matrix. (**Figure 1D**). In contrast, the negative control OP50-fed *C. elegans* showed no expanded intestinal lumen or intestinal epithelial cells filled with bacterial cell masses (data not shown). These results suggested that LGG could be attached to the *C. elegans* intestine, as determined by plate counting results and TEM images.

### LGG Enhanced Resistance to Pathogenic Infections in *C. elegans*

**Figure 2:**
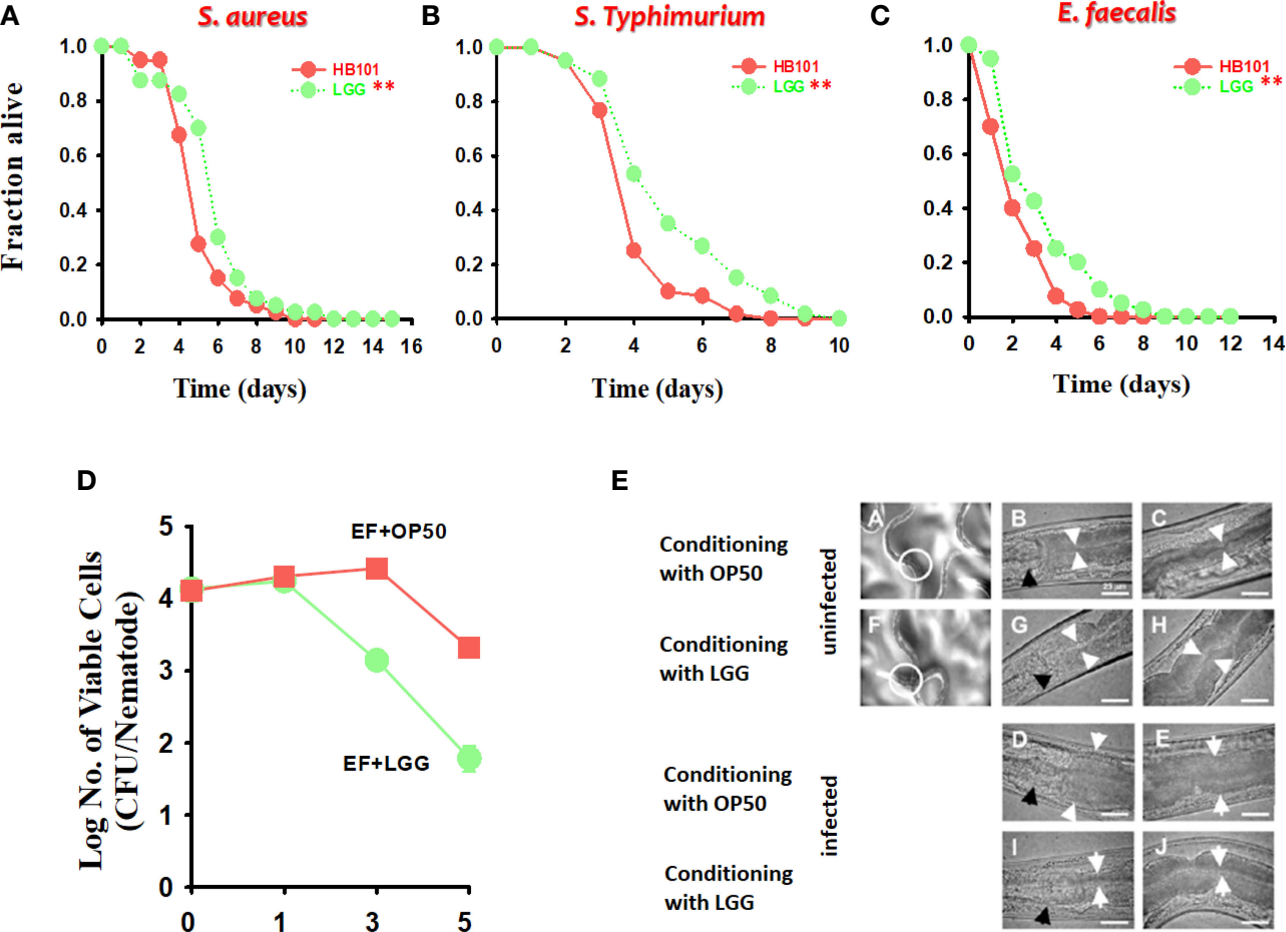
The effect of preconditioned LGG on the survival of *C. elegans* infected with both gram-positive and gram-negative pathogens. *C. elegans* killing assays of strains *fer-15;fem-1* infected with **(A)** *S. aureus* RN6390 or **(B)** *S. Typhimurium* SL1344 and *fer-15;ferm-1* and *glp-4* infected with **(C)** *E. faecalis* MMH594 after exposure to LGG for 24 h. The significance of differences between survival curves (Kaplan–Meier method) was determined using the log-rank test. *p < 0.05; **p < 0.01. **(D, E)** Bacterial burden (CFU/nematode) of *E. faecalis* in *fer-15;fem-1* nematodes after pre-exposure to OP50 or LGG. The white circle in A (exposed to OP50) and F (exposed to LGG) is the digestive tract and this area is for viewing at a higher magnification. White arrows indicate the borders of the intestinal lumen, and black arrows indicate the grinder organ. Scale bar, 25 μm.

In recent studies, *C. elegans* has been used as a laboratory animal model for screening for potential probiotic strains (Park et al., 2014; Zhou et al., 2014; Oh et al., 2018). In particular, *C. elegans* has a short life cycle and an inducible immune response resembling a significant part of the mammalian innate immune system, which is advantageous for studies on aging and immunity (Zhou et al., 2014; Park et al., 2018). We previously reported that probiotic strains, including *Lactobacillus* and *Bacillus*, improve lifespan by regulating the immune response of *C. elegans* (Kim and Mylonakis, 2012; Park et al., 2015; Park et al., 2018). We examined the immune response of *C. elegans* when exposed to LGG. Agar-based solid killing assays were performed with modifications according to a previously described method (Kim and Mylonakis, 2012; Park et al., 2018) to explore whether LGG augments the *C. elegans* defense response against pathogenic bacteria. *C. elegans* were transferred to LGG-plated plates for 24 h and then exposed to *S. aureus* RN6390 or *S. Typhimurium* SL1344 pathogenic bacteria, using a solid killing assay with the *fer-15;fem-1 C. elegans*. *C. elegans* exposed to LGG for 24 h clearly exhibited less susceptibility to pathogenic bacterial infection than the control strain OP50 (P = 0.0102 for *S. aureus*, P < 0.0001 for *S. Typhimurium*, respectively, compared to OP50) (**Figures 2A, B**).

Next, we evaluated whether LGG would colonize the *C. elegans* intestine, decreasing the number of pathogenic bacteria and prolonging *C. elegans* survival. To investigate this possibility, we evaluated the presence of cells by infecting *C. elegans* with *E. faecalis*, a pathogenic bacterium that colonizes the intestinal tract of the *C. elegans* to form a persistent lethal infection (Kim and Mylonakis, 2012) after 24 h of *C. elegans* exposure to LGG. First, we employed *C. elegans fer-15;fem-1* (Murphy et al., 2003) mutants and found that *C. elegans* exposed to LGG prolonged survival during *E. faecalis* infection (P = 0.0200 with *fer-15;fem-1* for LGG compared to *C. elegans* pre-exposed to OP50) (**Figure 2C**). Next, to measure the burden of *E. faecalis* in *C. elegans*, the *C. elegans* were exposed to *E. faecalis* infection after exposure to LGG and OP50 for 24 h. To remove surface bacteria, extensive washing was performed on BHI plates containing gentamicin as described previously (Kim and Mylonakis, 2012). In *C. elegans* pre-exposed to LGG or OP50, the number of *E. faecalis* cells in the *C. elegans* intestine was approximately 1.0 × 10^4^ CFU/*C. elegans* on days 0 and 1 after 24 h exposure to *E. faecalis*, and there was no difference between the two groups. However, in the case of *C. elegans* pre-exposed to LGG, the number of *E. faecalis* cells in the *C. elegans* intestine began to decrease from 3 days, and after approximately 5 days, the number of cells was confirmed to be approximately 8.0 × 10^1^ CFU/*C. elegans*. In the *C. elegans* pre-exposed to OP50, the number of *E. faecalis* cells in the *C. elegans* intestine was found to decrease from 5 days and colonize approximately 3.0 × 10^3^ CFU/*C. elegans* on day 5 (**Figure 2D**). Similar to the CFU results, in microscopic studies, *C. elegans* exposed to LGG showed a thin intestinal lumen after infection with *E. faecalis*, whereas the intestinal lumen of *C. elegans* pre-exposed to OP50 was distended by *E. faecalis* cells (**Figure 2E**). Consistent with our findings, LGG had a beneficial effect on the lifespan of *C. elegans*, colonizing the intestines of *C. elegans* (Zanni et al., 2015). Taken together, our results showed that LGG colonized the *C. elegans* intestine, which significantly inhibited the pathogenic bacteria in the small intestine of the *C. elegans* when the pathogen was infected, thereby increasing survival.

### LGG Regulates Specific Gene Transcriptions

**Figure 3:**
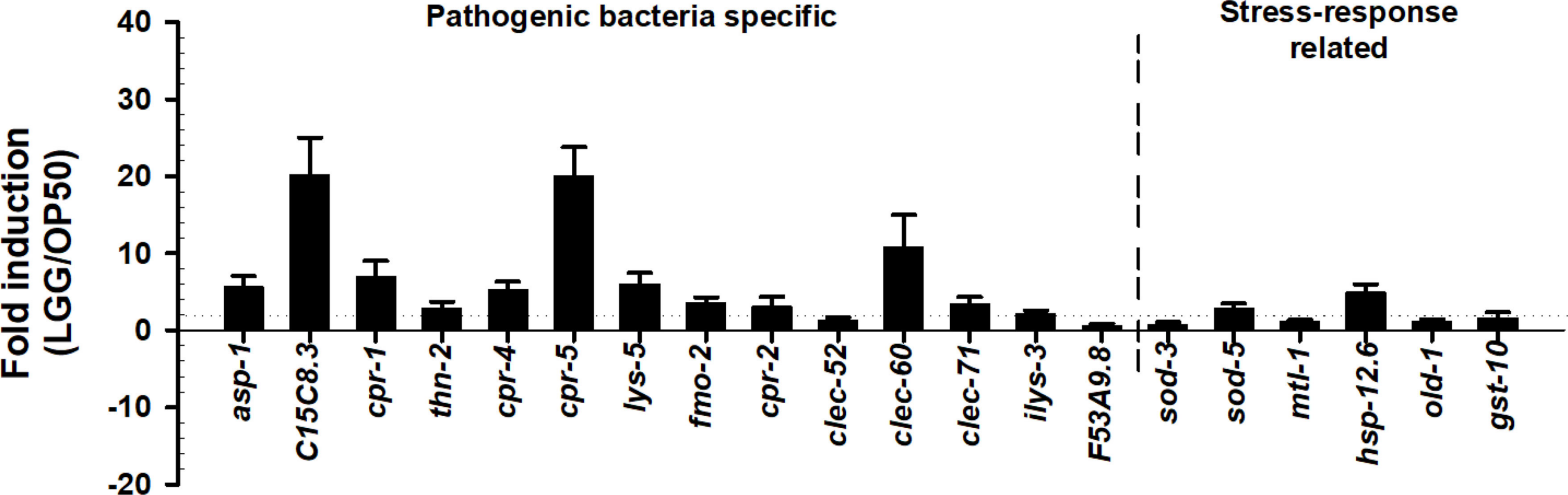
The alteration of the transcription of immune and stress related genes. Expression levels (LGG/OP50, normalized fold changes assessed by qRT-PCR analysis) of fourteen pathogenic bacteria specific immune response (*asp-1, C15C8.3, cpr-1, thn-2, cpr-4, lys-5, fmo-2, cpr-2, clec-52, clec-60, clec-71, ilys-3, F53A9.8*) and six stress response related genes (*sod-3, sod-5, mtl-1, hsp-12.6, old-1, gst-10*) in *C. elegans*. The dotted line shows 2-fold changes in LGG-induced gene expression compared to that of OP50-induced genes.

We determined the mechanism by which LGG is associated with the immunomodulation of *C. elegans*. Based on our findings that pre-exposure to LGG increases survival in *C. elegans* for pathogenic bacterial infections, we postulated that when pre-exposed to LGG, specific immune factors would be regulated in pathogenic bacterial infections of *C. elegans*. Based on previous studies (Kim and Mylonakis, 2012), we selected 14 genes from *C. elegans*, including *asp-1*, *C15C8.3* (encodes aspartyl proteases), *cpr-1*, *cpr-2*, *cpr-4* and *cpr-5* (encodes a cysteine protease), *clec-52*, *clec-60*, *clec-71* (encodes C-type lectins), *thn-2* (thaumatin/PR-5), *lys-5* (encodes a lysozyme), *fmo-2* (encodes a flavin-containing monooxygenase), *ilys-3* (encodes an invertebrate lysozyme), and *F53A9.8* (encodes a short His-rich protein), which are more than 4-fold increased by pathogenic bacteria. Additionally, we selected six genes related to stress in *C. elegans* (Hsu et al., 2003; Wang et al., 2010). Quantitative real-time PCR (qRT-PCR) was used to find that almost all selected genes in the *C. elegans* exposed to LGG were upregulated by 3.0-fold or more by LGG. Specifically, qRT-PCR results indicated that *asp-1* (5.7 ± 1.4-fold), *C15C8.3* (20.3 ± 4.8-fold), *cpr-1* (7.0 ± 2.1-fold), *cpr-4* (5.4 ± 0.94-fold), *cpr-5* (20.1 ± 3.7-fold), *lys-5* (6.1 ± 1.4-fold), *clec-60* (10.8 ± 4.2-fold), and *hsp-12.6* (5.0 ± 1.0-fold) transcription was induced dramatically by exposure to LGG (**Figure 3**). In contrast, four pathogenic bacteria-specific genes (*thn-2*, *clec-52*, *ilys-3*, *F53A9.8*) and five common stress-related genes (*sod-3*, *sod-5*, *mtl-1*, *old-1* and *gst-10*) were not affected by LGG exposure. These results are consistent with our results of the killing assay in which LGG exposure stimulated the resistance of *C. elegans* to infection by pathogenic bacteria (**Figure 2**). Our results are similar to studies showing that *C. elegans* survival was increased by the upregulation of immune-related genes when *C. elegans* exposed to *Lactobacillus* strains were infected with pathogenic bacteria (Kim and Mylonakis, 2012; Park et al., 2018). In particular, our group reported that antimicrobial genes, including *C15C8.3*, *cpr-1*, *cpr-5*, *lys-5*, and *clec-60*, were associated with the TIR-1, PMK-1, and BAR-1 signaling pathways (Kim and Mylonakis, 2012). Similar to previous studies (Kim and Mylonakis, 2012), *C15C8.3*, *cpr-1*, *cpr-5*, *lys-5*, and *clec-60* were also upregulated 6 times or more in this study. These results indicated that conditioning with LGG specifically stimulated the transcription of host defense genes associated with response to pathogen bacteria, thereby increasing lifespans.

### Expression Profiling Analysis for mRNA and miRNA of *C. elegans* Exposed to LGG

**Figure 4:**
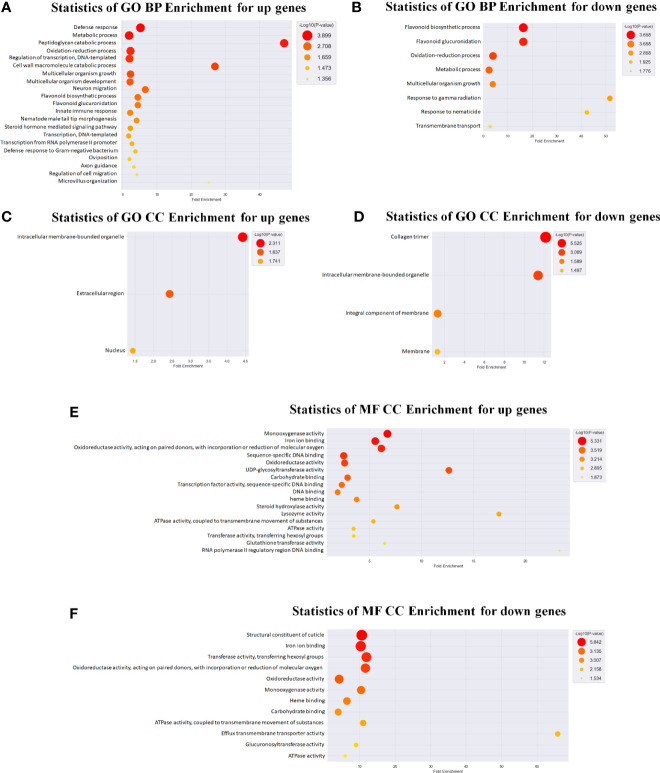
Gene ontology (GO) analysis in *C. elegans* exposed to LGG. GO analysis of differentially expressed genes was also performed. GO analysis was performed using the genes whose expression levels were changed by ≥3 folds compared with the control levels. Genes were classified based on DAVID (https://david.ncifcrf.gov/) and Medline databases (http://www.ncbi.nlm.nih.gov/). Data mining and graphic visualization were performed using ExDEGA (E-Biogen, Inc., Seoul, Korea). Dot plot shows the upregulated and downregulated genes GO terms (FDR <0.05) sorted by the most significant. The size and color of the dot shows the pathway enrichment significance. **(A, B)** Genes were classified into different hierarchical categories based on biological process, **(C, D)** cellular component and **(E, F)** molecular function.

**Figure 5:**
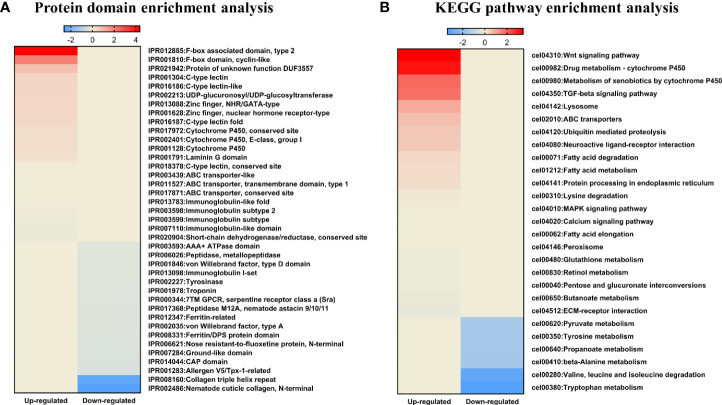
KEGG pathway and protein domain enrichment analyses in *C. elegans* exposed to LGG. Heatmap depicting abundance of selected protein domain and KEGG pathways. Different colors represent different expression level as determined by Fisher’s exact test p values (− log_10_). Red color represents up-regulated expression and blue color represents down-regulated expression. Each row denotes the categories of the protein domain and metabolic pathway. **(A)** Protein domain enrichment results of upregulated and downregulated proteins. **(B)** KEGG pathway enrichment results for upregulated and downregulated pathways.

We also used a microarray to identify changes in mRNA and miRNA expression in LGG-exposed *C. elegans*. Exposure to LGG for 24 h caused significant changes in transcript profiles in *C. elegans*. As a result, 261 downregulated and 1059 upregulated differentially expressed genes were screened out, while only 25 upregulated and 4 downregulated differentially expressed miRNAs had >2.0-fold changes compared with the control groups. For an in-depth understanding of the genome-wide distribution of *C. elegans* exposed to LGG, a comprehensive gene ontology (GO) enrichment analysis was performed. There were 21 upregulated and 8 downregulated GO terms belonging to biological processes (**Figures 4A, B**); 3 upregulated and 4 downregulated GO terms belonging to cellular components (**Figures 4C, D**); and 17 upregulated and 12 downregulated GO terms belonging to molecular functions (**Figures 4E, F**). Similar to the results of qRT-PCR, from the biological process-related GO enrichment analysis (**Figure 3**), the significantly upregulated GO terms against *C. elegans* immunity in LGG-exposed *C. elegans* included defense response, innate immune response, and defense response to gram-negative bacteria. Previously, other studies have reported that lactobacilli, including LGG, have positive effects on life and pathogenic infections by stimulating innate immune responses (Pinto et al., 2009; Kim and Mylonakis, 2012; Poupet et al., 2020). Additionally, the protein domain enrichment analyses in mRNA increased by 22 protein domains, including “F-box”, “cytochrome P450”, “C-type lectin” and “UDP-glucoronosyl/UDP-glucosyl transferase”, associated with immunity (**Figure 5A**) (Simonsen et al., 2012; Shimizu et al., 2021). The F-box protein targets virulent and pathogenic bacterial or viral proteins, contributing to its breakdown, and has been speculated to function as part of the *C. elegans* innate immune system (Thomas, 2006). C-type lectins have been reported to be involved in serum glycoprotein homeostasis, pathogen detection, and initiation of an immune response (Mayer et al., 2017). UDP-glucuronyl transferase (UGT) and cytochrome P450 are involved in chemical detoxification and innate immune responses (Simonsen et al., 2012; Tan et al., 2020). Tan et al. reported that C-type lectin, UDP-glucuronyl transferase, and F-box A protein were downregulated when infected with pathogenic *E. coli*, and they were upregulated in probiotic treatment (Tan et al., 2020). Therefore, exposure to LGG can improve the innate immune response of the *C. elegans* while prolonging its lifespan.

In addition, we used DAVID (version 6.8) (Harvald et al., 2017) for mRNAs that changed after exposure to LGG to identify the enriched KEGG (Kyoto Encyclopedia of Genes and Genomes) pathways within each cluster (**Figure 5B**). Among the enriched KEGG pathways, we identified several immune response pathways, including the MAPK signaling pathway, Wnt signaling pathway and TGF-beta signaling pathway. The MAPK pathway is the innate immune system, the first line of defense against environmental microbial infections, and is evolutionarily conserved between vertebrates and invertebrates (Sun et al., 2016). Other studies found that the innate immune response of *C. elegans* exposed to *Lactobacillus* spp. were mediated by the MAPK signaling pathway (Kim and Mylonakis, 2012; Park et al., 2018). In another study, lactoferrin uptake was reported to modulate several genes that stimulate the immune function of *C. elegans* through induction of the TGF-β and Wnt signaling pathways and encode molecular effectors involved in the innate immune response (Nicholas and Hodgkin, 2004; Martorell et al., 2017). TGF-β pathway function is associated with protection against pathogenic bacteria, axonal pathfinding, body size/male tail development, and dauer formation (Patterson and Padgett, 2000). The Wnt signaling pathway is formed by highly conserved secreted signaling molecules that regulate cell-cell interactions, cell proliferation and differentiation during embryogenesis and affect the development of the central nervous system in vertebrates (Kim et al., 2013; Rosso and Inestrosa, 2013). Therefore, exposure to LGG was found to be related to various immune responses of *C. elegans*, thereby affecting the protective effect against pathogenic bacteria and the increase in lifespan.

Next, we investigated on the miRNAs target prediction using TargetScan (http://www.targetscan.org/). Since miRNA regulates gene expression through degradation or translation inhibition of target mRNA (Bushati and Cohen, 2007), we finally investigated mRNA whose expression correlates negatively with miRNA expression. Therefore, we identified 20 predicted target genes that were downregulated in 25 upregulated miRNAs and 1 target gene that was upregulated in 4 downregulated miRNAs (**Table 1**). In particular, we identified 3 miRNAs (*cel-miR-796*, *cel-miR-34-3p*, *cel-miR-239a-5p*) that regulate more than 4 target genes. Additionally, Liu et al. reported that loss of *miR-34* accelerates aging and brain damage in *Drosophila* (Liu et al., 2012). These results suggest that miR-34 may play a crucial role in regulating the immune response of *C. elegans* by indirectly regulating immune-related genes instead of directly.

**Table 1:** List of miRNAs that were significantly regulated in *C. elegans* exposed to LGG.

NO.	MicroRNAs	Accession	FC	Potential target mRNAs
1	*cel-miR-43-3p*	MIMAT0000014	6.340	*alh-9*
2	*cel-miR-1823*	MIMAT0006590	5.486	
3	*cel-miR-796*	MIMAT0004231	5.210	*cnc-2, acs-1, fkh-2, grl-4, alh-9, exc-9*
4	*cel-miR-5546-3p*	MIMAT0022184	5.135	
5	*cel-miR-42-3p*	MIMAT0000013	3.789	*tnt-2*
6	*cel-miR-76-3p*	MIMAT0000048	2.938	
7	*cel-miR-5550-3p*	MIMAT0022192	2.567	
8	*cel-miR-795-3p*	MIMAT0020353	2.567	*nhr-43, col-91, lin-41*
9	*cel-miR-1832a*	MIMAT0006774	2.527	
10	*cel-miR-41-3p*	MIMAT0000012	2.467	*tnt-2*
11	*cel-miR-34-3p*	MIMAT0015093	2.399	*cpg-9, col-101, gsnl-1, col-181*
12	*cel-miR-356b-3p*	MIMAT0022312	2.379	
13	*cel-miR-239a-5p*	MIMAT0000294	2.344	*tyr-1, dpy-6*
14	*cel-miR-55-5p*	MIMAT0020313	2.312	*lon-1*
15	*cel-miR-242*	MIMAT0000298	2.265	
16	*cel-miR-57-3p*	MIMAT0020314	2.254	
17	*cel-miR-1830-3p*	MIMAT0020778	2.254	
18	*cel-miR-38-3p*	MIMAT0000009	2.254	*tnt-2*
19	*cel-miR-38-5p*	MIMAT0020305	2.193	
20	*cel-miR-784-3p*	MIMAT0020347	2.165	
21	*cel-miR-42-5p*	MIMAT0015095	2.093	
22	*cel-miR-2209a-3p*	MIMAT0011432	2.083	*grl-4, cnc-4, tag-153, grsp-1, cnc-3*
23	*cel-miR-2214-5p*	MIMAT0011443	2.036	
24	*cel-miR-2209c-3p*	MIMAT0011434	2.020	
25	*cel-miR-358-3p*	MIMAT0000700	2.012	
26	*cel-miR-70-3p*	MIMAT0000042	0.499	*csq-1*
27	*cel-miR-40-5p*	MIMAT0020307	0.497	
28	*cel-miR-265*	MIMAT0000321	0.476	
29	*cel-miR-249-5p*	MIMAT0020338	0.461	

### LGG Prolongs the Lifespans by Increasing miR-34 Expression in *C. elegans*

**Figure 6:**
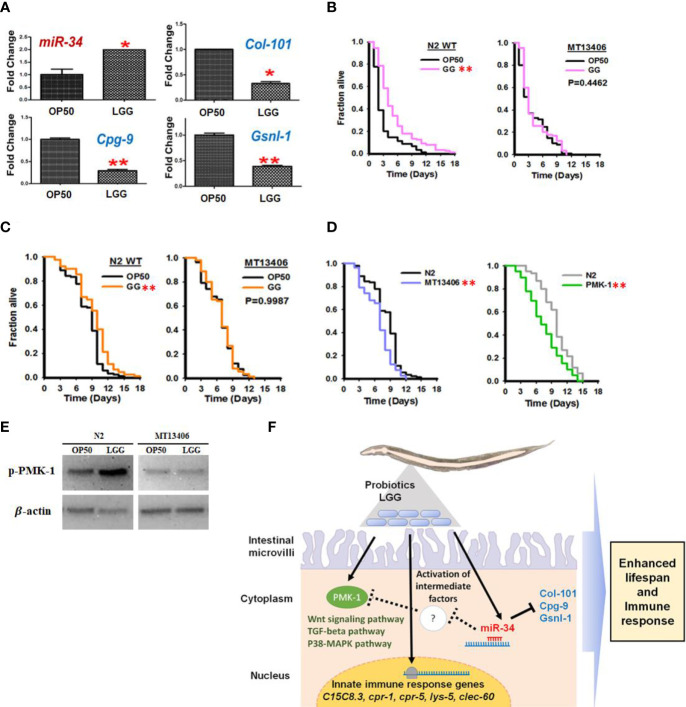
Stimulation of miR-34 and PMK-1 pathways in *C. elegans* by ingestion of LGG. **(A)** *C. elegans* exposed to LGG upregulated *miR-34* and inhibited its putative target genes *col-101*, *cpg-9* and *gsnl-1* which was selected among down regulated genes from Affymetrix GeneChip_miRNA array data. **(B)** The killing assay of N2 wild type and *miR-34* deletion mutant (MT13406) exposed to OP50 or LGG against to pathogenic bacteria (*S. aureus*). **(C)** The longevity assay of N2 wild type and *miR-34* mutant (MT13406) exposed to OP50 or LGG **(D)** The longevity assay of *pmk-1* deletion mutant (*km25)IV* exposed to OP50 or LGG. The values are *P<0.05; **P<0.01 compared with OP50. **(E)** Protein expression of phosphorylated PMK-1 in LGG or OP50 exposed to miR-34 mutant / N2 WT assessed by Western blotting. β-actin was used as loading control. **(F)** Potential mechanism of LGG exposure and their functions in regulating innate immunity in *C. elegans* through stimulating miR-34 and PMK-1. Intermediate factors including may exist between miR-34 activation and PMK-1 pathway.

Based on our microarray results showing that LGG regulates *miR-34* in *C. elegans* and prolongs its life, we revalidated the results using qRT-PCR and loss-of-function *miR-34 C. elegans* mutant. We analyzed the TargetScan database (Krek et al., 2005) for predicting target sequences of *miR-34*. The putative *miR-34* targets include collagen genes (*col-101*), chondroitin proteoglycan (*cpg-9*), and gelsolin-like protein 1 (*gsnl-1*). The expression of *miR-34* was increased in *C. elegans* exposed to LGG, and the putative target genes of *miR-34*, *col-101*, *cpg-9* and *gsnl-1* were significantly downregulated by LGG (**Figure 6A**). In addition, in N2 wild-type *C. elegans* exposed to LGG, survival in pathogenic bacteria (*S. aureus*) was prolonged, but exposure to LGG in *C. elegans* with deleted *miR-34* did not affect survival (**Figure 6B**). In the aging assay, survival was extended in N2 wild *C. elegans* exposed to LGG, but exposure to LGG did not affect survival in the *mir-34* mutant strain (**Figure 6C**). In particular, aging assays after exposure to LGG were performed using mutations that deleted the *pmk-1* gene, which has been reported to play a role in regulating longevity and innate immunity genes. As a result, the *pmk-1* mutant showed a shortened lifespan similar to the *miR-34* mutant (**Figure 6D**). Next, we determined the level of phosphorylated PMK-1 using a specific antibody that recognizes the phosphorylated form of PMK-1 to determine whether LGG affects the activity of PMK-1. While the levels of phosphorylation of PMK-1 were critically upregulated by LGG in N2 wild *C. elegans*, this indicates that phosphorylation levels were not regulated by exposure to LGG in miR-34 mutant *C. elegans* (**Figure 6E**). We found that LGG exposure affects miRNAs in *C. elegans*, resulting in enhanced immunity and longevity. *miR-34* was associated with lifespan and the onset of age-related diseases in model organisms, but the direction and mechanism underlying its effects are still unclear (Pinto et al., 2018). Previous studies have shown that *miR-34* significantly prolongs life in removed mutations (Pinto et al., 2018), but other studies have reported findings that either did not affect survival (De Lencastre et al., 2010) or even the reverse, prolonged life (Liu et al., 2012). We found that when *C. elegans* was exposed to LGG, it upregulated *miR-34* and affected lifespan (**Figures 6A–C**). However, mutations with *miR-34* removed did not affect lifespan even when exposed to LGG (**Figures 6B–D**). Importantly, LGG has been shown to affect the PMK-1 pathway involved in the innate immunity of *C. elegans* (**Figure 6E**) (Park et al., 2018). Our group has previously identified that *Lactobacillus* strains enhance susceptibility to infection of pathogenic bacteria of *C. elegans via* the PMK-1 pathway (Kim and Mylonakis, 2012; Park et al., 2018). Zhou et al. also reported that *Lacticaseibacillu zeae* regulates *C. elegans* signaling through the p38 MAPK pathway to control the production of antimicrobial peptides and defense molecules to combat ETEC infection (Zhou et al., 2018). Similarly, our results show that LGG effectively stimulates the PMK-1 signaling pathway in *C. elegans* thereby combating pathogenic bacteria. Xiong et al. reported that *miR-34* is related to innate immunity (Xiong et al., 2016). Similarly, our study supports that *miR-34* affects PMK-1 as there is no significant change in PMK-1 expression in *miR-34* mutant *C. elegans* by LGG exposure. Thus, we found that *miR-34* stimulated by LGG exposure of *C. elegans* activates the PMK-1 pathway by downregulating a candidate gene (**Figure 6F**).

## Conclusions

In this study, we have demonstrated that LGG has a beneficial effect on *C. elegans* lifespan and pathogenic bacterial infections. We confirmed that LGG protects against pathogen infection and prolongs lifespan by enhancing MAPK signaling pathway, Wnt signaling pathway and TGF-β signaling pathway of *C. elegans*. Our study also confirmed that antimicrobial genes were significantly increased by LGG. In addition, it was confirmed that the expression of *miR-34* was increased due to LGG, and they could potentially activate the PMK-1. Taken together, our results confirm another potential of LGG probiotics for prevention of aging and metabolic diseases in functional foods. In addition, our study enhances the immune-modulated miRNA repertoire in host and provides new insights into the interaction between innate immunity. We concluded that LGG stimulated miRNAs in *C. elegans* to enhance resistance to pathogen infection and prolong lifespan.

## Data Availability Statement

The original contributions presented in the study are included in the article/**Supplementary Material**. Further inquiries can be directed to the corresponding authors.

## Author Contributions

BY, YK, and SO conceived and designed the experiments. BY, SR, MK, JL, JY, YK, and SO performed the experiments. BY, SR, MK, JL, JY, YK, and SO analyzed the data. BY, YK, and SO wrote the paper. All authors contributed to the article and approved the submitted version.

## Funding

This research was supported by a National Research Foundation of Korea Grant, funded by the Korean government (MEST) (NRF-2018R1D1A3B07050304 to SO and 2021R1A2C3011051 to YK).

## Conflict of Interest

The authors declare that the research was conducted in the absence of any commercial or financial relationships that could be construed as a potential conflict of interest.

## Publisher’s Note

All claims expressed in this article are solely those of the authors and do not necessarily represent those of their affiliated organizations, or those of the publisher, the editors and the reviewers. Any product that may be evaluated in this article, or claim that may be made by its manufacturer, is not guaranteed or endorsed by the publisher.
